# BS-SNPer: SNP calling in bisulfite-seq data

**DOI:** 10.1093/bioinformatics/btv507

**Published:** 2015-08-28

**Authors:** Shengjie Gao, Dan Zou, Likai Mao, Huayu Liu, Pengfei Song, Youguo Chen, Shancen Zhao, Changduo Gao, Xiangchun Li, Zhibo Gao, Xiaodong Fang, Huanming Yang, Torben F. Ørntoft, Karina D. Sørensen, Lars Bolund

**Affiliations:** ^1^Department of Biomedicine, Aarhus University, Aarhus, Denmark,; ^2^BGI Co. Ltd., Shenzhen 518083, China,; ^3^BioInformatics Research Center (BIRC), Aarhus University, Aarhus 8000, Denmark,; ^4^School of Computer, National University of Defense Technology, Changsha 410073, China,; ^5^Genomic Biology Laboratory, QIMR Berghofer Medical Research Institute, Brisbane, Australia,; ^6^Departments of Biostatistics and Informatics, University of Colorado-Anschutz Medical Campus, Denver 80204, USA; ^7^The Fourth People's Hospital of Shenzhen (Futian hospital), Shenzhen, 518033, China,; ^8^Department of Obsterics and Gynecology,the First Hospital Affiliated to Suzhou University, Jiangsu Suzhou 215006,China,; ^9^College of Information Engineering, Qingdao University, Qingdao 266071, China and; ^10^Department of Molecular Medicine, Aarhus University Hospital, Aarhus 8000, Denmark

## Abstract

Summary: Sodium bisulfite conversion followed by sequencing (BS-Seq, such as whole genome bisulfite sequencing or reduced representation bisulfite sequencing) has become popular for studying human epigenetic profiles. Identifying single nucleotide polymorphisms (SNPs) is important for quantification of methylation levels and for study of allele-specific epigenetic events such as imprinting. However, SNP calling in such data is complex and time consuming. Here, we present an ultrafast and memory-efficient package named BS-SNPer for the exploration of SNP sites from BS-Seq data. Compared with Bis-SNP, a popular BS-Seq specific SNP caller, BS-SNPer is over 100 times faster and uses less memory. BS-SNPer also offers higher sensitivity and specificity compared with existing methods.

**Availability and implementation**: BS-SNPer is written in C++ and Perl, and is freely available at https://github.com/hellbelly/BS-Snper.

**Contact**: bolund@biomed.au.dk, kdso@clin.au.dk or orntoft@ki.au.dk

**Supplementary information:** Supplementary data are available at *Bioinformatics* online.

## 1 Introduction

Sodium bisulfite conversion followed by sequencing (BS-Seq) has become popular for studying human epigenetic profiles (Laird, 2010). Whole genome bisulfite sequencing detects 90% of methylation events and SNPs in the nuclear DNA (Lister *et al.*, 2009). However, its cost remains too high for most laboratories. Reduced representation bisulfite sequencing (RRBS) is a more efficient and economical way to monitor genome-wide promoter and CpG island methylation status (Laird, 2010) and it has become a popular method to investigate epigenetic changes in clinical tissue samples (Berman *et al.*, 2012; Hansen *et al.*, 2011).

SNP identification is important for identification of allele-specific epigenetic events such as, gamete specific and genetic imprinting (Reik and Walter, 2001; Wilkins, 2005). However, SNP calling from BS-Seq data has been shown to be complicated. One problem is that reads from two genomic strands are not complementary at methylated loci. Two methods were widely used to solve this problem. One is to align the reads in a three-letter space; the other is a wildcard algorithm which accounts for the C/T conversions. Many software packages have been developed based on these two methods. Bismark (Krueger and Andrews, 2011), MethylCoder (Pedersen *et al.*, 2011), BRAT-BW (Harris *et al.*, 2012) and BS Seeker (Chen *et al.*, 2010) are based on the Burrows–Wheeler transform. Bismark and BS Seeker use Bowtie (Langmead and Salzberg, 2012) to align the reads in a three-letter space. BSMAP (Xi and Li, 2009) uses a wildcard algorithm.

Another problem is that true C/T SNPs in samples cannot be distinguished from C/T substitutions caused by bisulfite conversion and, thus, could be misidentified as unmethylated Cs (Liu *et al.*, 2012). Given that over two-thirds of all SNPs occur in CpG context (Tomso and Bell, 2003), sequence variations need to be addressed as an important error source. After alignment, real methylation status could be recovered, because a C/T SNP in Watson strand and G/A SNP in Crick strand should be interpreted as unmethylated cytosine variation (see Supplementary Fig. S1). Thus, Watson and Crick strands should be independently treated to reduce such errors.

To our knowledge, Bis-SNP is a popular BS-Seq data based SNP calling tool. Another BS-Seq specific SNP caller, MethylExtract, is slightly faster than Bis-SNP (Barturen *et al.*, 2013). However, Bis-SNP performs better in sensitive and accuracy. Here, we present BS-SNPer, a program for BS-Seq variation detection from alignments in standard BAM/SAM format (Li *et al.*, 2009). We implemented a novel algorithm (called ‘dynamic matrix algorithm’, see text below) and approximate Bayesian modeling to improve the performance. Using published RRBS data, BS-SNPer showed higher specificity and sensitivity with lower memory requirement and was over 100 times faster than Bis-SNP.

## 2 Methods

Two steps are implemented to obtain the final SNP set. In the first step, a candidate SNP set is obtained from alignments, usually in the BAM/SAM format, using a novel method ‘dynamic matrix algorithm’. In the second step, the candidate set was converted to the final SNP set using Bayes model, considering alignment quality and read support.

### Step 1. Dynamic matrix algorithm

Alignments are filtered based on sequencing quality, mapping quality and mismatch rates. Mutations are removed if their frequencies are lower than a certain threshold (default 0.1). The formulae to calculate the frequencies are listed in Supplementary Table S1. For the remaining candidate SNP set, positions, reference bases, the numbers of supporting reads and average sequencing quality for all four bases in both Watson and Crick strands are recorded. In order to improve memory and computation efficiency, these data are dynamically allocated and freed for each chromosome. For each position in a chromosome, the data are stored in the form of a vector; thus the data of a chromosome are stored in a matrix. The content and size of the matrix change with the chromosomes. The method is thus called ‘dynamic matrix algorithm’.

### Step 2. Approximate Bayesian modeling

In brief, the Bayesian inference of each genotype is based on its posterior distributions, *P*(*G*|*D*), using Bayes’ formula. The posterior distribution is built upon two components: the prior distribution of each genotype *P*(*G*), and the likelihood *P*(*D*|*G*), which is the probability of observing reads *D* given genotype *G.* For the prior *P*(*G*), we referred to the model of SOAPsnp (Li *et al.*, 2009). We observed that, when sequencing depth was higher than 10, the choice of the prior had no large effects. The likelihood, which represents the error rates caused by multiple sources, is calculated by the formula *P*(*D*|*g* = *G*) = $\prod_{i=1}^{n}P(Di|g=G)$ for multiple independent samples *D_i_*, where *i* = 1, 2,…, *n*, and *n* is the number of reads. We used average error rate instead of full evaluation in error rate, which increases the modeling speed. The genotype with largest probability is recognized as the final SNP. See Supplementary Text for more details.

## 3 Results

Our previous RRBS data (Huang *et al.*, 2014) were used to test the performance of BS-SNPer and to compare it with Bis-SNP. The data comprise one para-normal sample (below called ‘Normal’; normal tissue adjacent to cancer tissue at a distance of at least 5 cm) and three cancer samples, i.e. primary renal cell carcinomas (pRCC), local invasion of the vena cava (IVC) and distant metastasis to the brain (MB) tissues from a patient with metastatic clear cell renal cell carcinoma. Whole exome sequencing data in the same work (Huang *et al.*, 2014) were employed to assess the performance of SNP calling.

All reads of four samples were aligned to the GRCh37 assembly (hg19) of the human genome using the program BSMAP with options ‘-z 64 -p 12 -s 16 -v 10 -q 2’. The alignments were then fed into both BS-SNPer, MethylExtract and Bis-SNP under same conditions. We evaluated the algorithms on a system equipped with a six-core Intel Xeon E5650 2.66 GHz processor and 32 GB memory. The system runs on 64-bit Red Hat Enterprise Linux 4.1. The minimal running time of four samples of MethylExtract and Bis-SNP was 6 and 20 h, respectively, whereas BS-SNPer only used around 10 min for each sample. Compared with Bis-SNP, the increase in speed was more than 100 times in all four cases, including normal tissue (Normal), pRCC, local IVC, distant MB, of our published data (Huang *et al.*, 2014). The increase in speed was not at the cost of memory or accuracy. The maximal memory usage of BS-SNPer was 8 GB, whereas MethylExtract and Bis-SNP used 10 GB for all cases, respectively. BS-SNPer also showed higher sensitivity and specificity for all four samples (Supplementary Table S2). For example, in Normal tissue, 2730 SNPs were detected by BS-SNPer, among which 2335 were validated by exome data [false positive rate (FPR) 14.47%]. Bis-SNP detected 3483 SNPs, while 2011 of them were validated (FPR 42.26%; MethylExtract 39.93%). As exome sequencing detected 2873 SNPs for this sample, false negative rate of BS-SNPer was 18.73%, whereas that of Bis-SNP and MethylExtract was 30% and 57.01%, respectively (Supplementary Table S2).

In conclusion, based on a dynamic matrix algorithm and Bayesian statistical framework, we present BS-SNPer, a SNP calling tool for BS-Seq data. BS-SNPer provides high performance in terms of speed, memory usage, accuracy and sensitivity.

## Supplementary Material

Supplementary Data

## References

[btv507-B1] Barturen G. (2013) MethylExtract: high-quality methylation maps and SNV calling from whole genome bisulfite sequencing data. F1000Res., 2, 217. 24627790 10.12688/f1000research.2-217.v1 PMC3938178

[btv507-B2] Berman B.P. (2012) Regions of focal DNA hypermethylation and long-range hypomethylation in colorectal cancer coincide with nuclear lamina-associated domains. Nat. Genet., 44, 40–46. 22120008 10.1038/ng.969 PMC4309644

[btv507-B3] Chen P.Y. (2010) BS Seeker: precise mapping for bisulfite sequencing. BMC Bioinformatics, 11, 203. 20416082 10.1186/1471-2105-11-203 PMC2871274

[btv507-B4] Hansen K.D. (2011) Increased methylation variation in epigenetic domains across cancer types. Nat. Genet., 43, 768–775. 21706001 10.1038/ng.865 PMC3145050

[btv507-B5] Harris E.Y. (2012) BRAT-BW: efficient and accurate mapping of bisulfite-treated reads. Bioinformatics, 28, 1795–1796. 22563065 10.1093/bioinformatics/bts264 PMC3381974

[btv507-B6] Huang Y. (2014) Multilayered molecular profiling supported the monoclonal origin of metastatic renal cell carcinoma. Int. J. Cancer, 135, 78–87. 24310851 10.1002/ijc.28654

[btv507-B7] Krueger F. Andrews S.R. (2011) Bismark: a flexible aligner and methylation caller for bisulfite-seq applications. Bioinformatics, 27, 1571–1572. 21493656 10.1093/bioinformatics/btr167 PMC3102221

[btv507-B8] Laird P.W. (2010) Principles and challenges of genomewide DNA methylation analysis. Nat. Rev. Genet., 11, 191–203. 20125086 10.1038/nrg2732

[btv507-B9] Langmead B. Salzberg S.L. (2012) Fast gapped-read alignment with Bowtie 2, Nat. Methods, 9, 357–359. 10.1038/nmeth.1923 PMC3322381 22388286

[btv507-B10] Li H. (2009) The Sequence Alignment/Map format and SAMtools. Bioinformatics, 25, 2078–2079. 19505943 10.1093/bioinformatics/btp352 PMC2723002

[btv507-B11] Li R. (2009) SNP detection for massively parallel whole-genome resequencing. Genome Res., 19, 1124–1132. 19420381 10.1101/gr.088013.108 PMC2694485

[btv507-B12] Lister R. (2009) Human DNA methylomes at base resolution show widespread epigenomic differences. Nature, 462, 315–322. 19829295 10.1038/nature08514 PMC2857523

[btv507-B13] Liu Y. (2012) Bis-SNP: Combined DNA methylation and SNP calling for Bisulfite-seq data. Genome Biol., 13, R61. 22784381 10.1186/gb-2012-13-7-r61 PMC3491382

[btv507-B14] Pedersen B. (2011) MethylCoder: software pipeline for bisulfite-treated sequences. Bioinformatics, 27, 2435–2436. 21724594 10.1093/bioinformatics/btr394 PMC3157921

[btv507-B15] Reik W. Walter J. (2001) Genomic imprinting: parental influence on the genome. Nat. Rev. Genet., 2, 21–32. 11253064 10.1038/35047554

[btv507-B16] Tomso D.J. Bell D.A. (2003) Sequence context at human single nucleotide polymorphisms: overrepresentation of CpG dinucleotide at polymorphic sites and suppression of variation in CpG islands. J. Mol. Biol., 327, 303–308. 12628237 10.1016/s0022-2836(03)00120-7

[btv507-B17] Wilkins J.F. (2005) Genomic imprinting and methylation: epigenetic canalization and conflict. Trends Genet., 21, 356–365. 15922835 10.1016/j.tig.2005.04.005

[btv507-B18] Xi Y. Li W. (2009) BSMAP: whole genome bisulfite sequence MAPping program. BMC Bioinformatics, 10, 232. 19635165 10.1186/1471-2105-10-232 PMC2724425

