# Supplementary material for: BS-SNPer: SNP calling in bisulfite-seq data
Source: Bioinformatics. 2015 Aug 28;31(24):4006–8. doi: 10.1093/bioinformatics/btv507 (PMC4673977; doi:10.1093/bioinformatics/btv507)
Supplement: Supplementary Data [file supp_31_24_4006__index.html]

BS-SNPer: SNP calling in Bisulfite-seq data — BS-SNPer: SNP calling in bisulfite-seq data — BS-SNPer: SNP calling in bisulfite-seq data — Supplementary Data 

# BS-SNPer: SNP calling in bisulfite-seq data

## Supplementary Data

files

- Supplementary Data - doc file
