# Supplementary material for: BS-SNPer: SNP calling in bisulfite-seq data
Source: Bioinformatics. 2015 Aug 28;31(24):4006–8. doi: 10.1093/bioinformatics/btv507 (PMC4673977; doi:10.1093/bioinformatics/btv507)
Supplement: Supplementary Data [file supp_btv507_Supplementary.doc]

**Table of Contents**

- Figure S1
- Table S1
- Table S2
- Supplementary Text

**
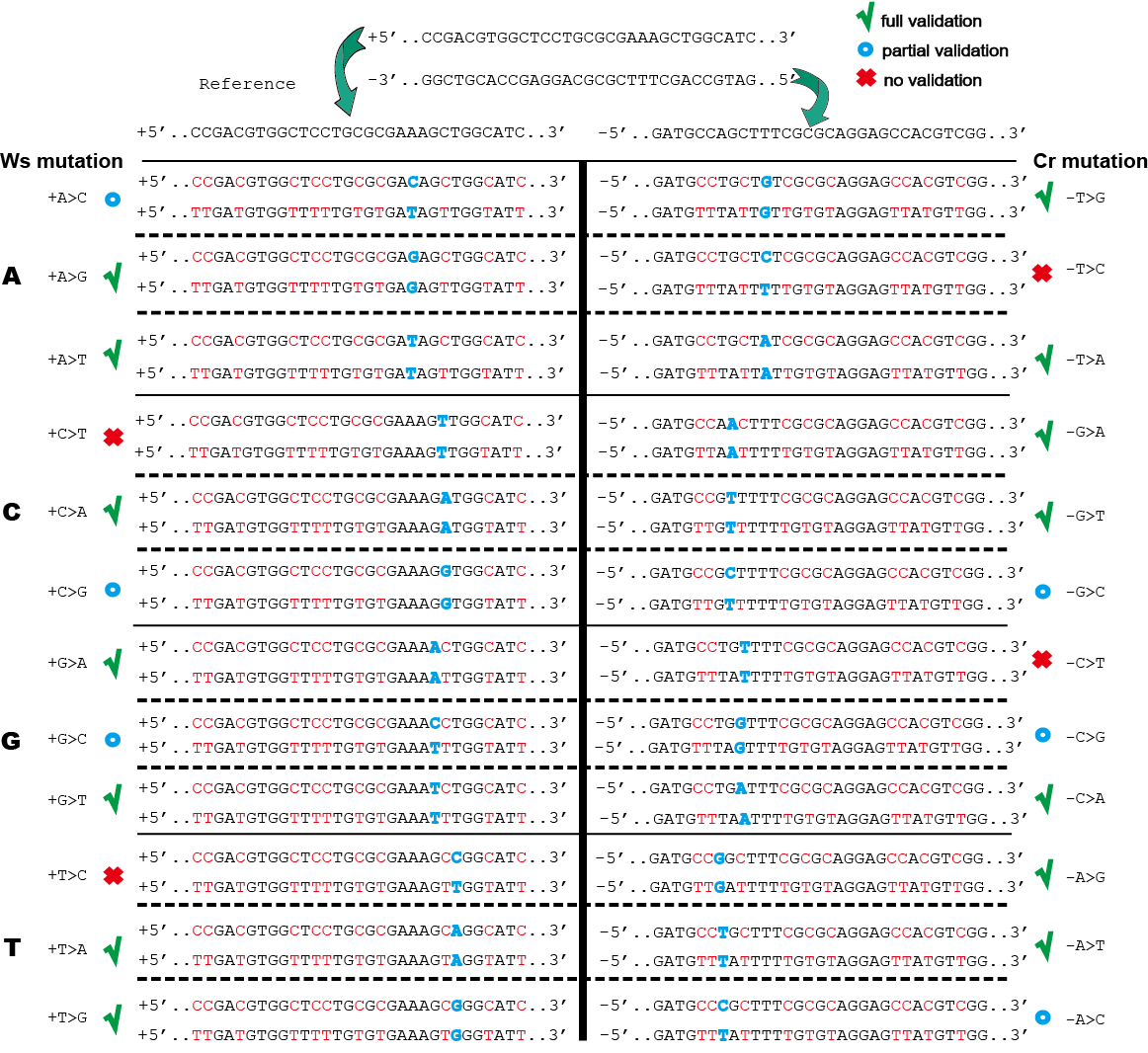
**

**Figure S1. Illustration of all genotypes before and after bisulfite treatment.** The left and right panels show plus and minus strands, respectively. Each row shows one of all 12 possible mutations. 12 mutations are divided into 4 groups with same starting nucleotide in same group. Solid horizontal lines separate these 4 groups. The 3 mutations of any starting nucleotide are separated by dashed horizontal lines. The upper and lower sequences in each row show genotypes before and after bisulfite treatment, respectively. Red letters show unmethylated C sites (thus affected by bisulfite treatment). Mutations are shown in blue letters. Original reference sequences of both strands are shown at the top of the figure. Considering strands shown in left and right panels, there are 24 mutations in total. For each of the 24 mutations, there are 3 status of validation based on whether the mutation could be validated only by this read or need complementary read as well: full validation (marked in green hooks, need this read only), no validation (marked in red crosses, cannot be validated) and partial validation (marked in blue circles, need complementary read). The genotypes with no validation cannot be validated because they do not appear polymorphic after conversion. Ws: Watson strand; Cr: Crick strand.

**Table S1. Formulae for the calculation of mutation frequencies.**

| **Type of mutation** | **Mutation frequency** |
| --- | --- |
| A>T | T reads / Total reads |
| A>C | (C+WsT) reads / Total reads |
| A>G | WsG reads / (WsG+WsA) reads |
| T>A | A reads / Total reads |
| T>C | CrC reads / (CrT+CrC) reads |
| T>G | (G+CrA) reads / Total reads |
| C>A | A reads / Total reads |
| C>T | CrT reads / (CrC+CrT) reads |
| C>G | (G+CrA) reads / Total reads |
| G>A | WsA reads / (WsA+WsG) reads |
| G>T | T reads / Total reads |
| G>C | (WsT+C) reads / Total reads |

Abbreviations: WsN reads: The number of reads supporting base N in Watson strand; CrN: The number of reads supporting base N in Crick strand; N reads: The number of reads supporting base N in both strands.

**Table S2. Comparison among BS-SNPer, MethylExtract and Bis-SNP using a real data set (ccRCC).**

|  | **Normal** | | | **pRCC** | | | **IVC** | | | **MB** | | | |
| --- | --- | --- | --- | --- | --- | --- | --- | --- | --- | --- | --- | --- | --- |
|  | **BS^*^** | **ME** | **Bis-SNP** | **BS** | **ME** | **Bis-SNP** | **BS** | **ME** | **Bis-SNP** | **BS** | | **ME** | **Bis-SNP** |
| **# Exome SNPs** | 2873 | 2873 | 2873 | 3401 | 3401 | 3401 | 2995 | 2995 | 2995 | 2966 | | 2966 | 2966 |
| **# Called SNPs** | 2730 | 2056 | 3483 | 3106 | 2998 | 3308 | 2878 | 2445 | 3906 | 2871 | | 2266 | 4355 |
| **# Validated SNPs** | 2335 | 1235 | 2011 | 2230 | 1397 | 2195 | 2270 | 1298 | 2145 | 2261 | | 1250 | 2176 |
| **FNR** | 18.73% | 57.01% | 30.00% | 34.43% | 58.92% | 35.46% | 24.21% | 56.66% | 28.38% | 23.77% | 57.86% | | 26.64% |
| **FPR** | 14.47% | 39.93% | 42.26% | 28.20% | 53.40% | 33.65% | 21.13% | 46.91% | 45.08% | 21.25% | 44.84% | | 50.03% |

Abbreviations: Exome SNPs: SNPs detected in target regions using exome sequencing data, which were used as standard. Normal: Para-normal tissue; pRCC: Primary renal cell carcinomas; IVC: Local invasion of the vena cava; MB: Distant metastasis to the brain; ccRCC: Clear cell renal cell carcinoma; SNPer: BS-SNPer; ME: MethylExtract; BS: BS-SNPer.

^*^Except for BS-SNPer, we used default settings for all programs. The command line used in BS-SNPer is as below.

perl BS-Snper.pl --fa hg19.fa --input outfile.sort.bam --output tempoutput --methoutput Meth.out --minhetfreq 0.1 --minhomfreq 0.85 --minquali 15 --mincover 10 --maxcover 1000 --minread2 2 --errorate 0.02 >SNP.out

**Supplementary Text**

METHODS

The algorithms of BS-SNPer

Two steps are implemented to obtain the final SNP set. In the first step, a candidate SNP set is obtained from alignments, usually in the BAM/SAM format, using dynamic matrix algorithm. In the second step, the candidate set was converted to the final SNP set using approximate Bayes model, considering alignment quality and read support.

*Step 1. Dynamic matrix algorithm.* Sorted alignments in BAM format are used as input. The alignments are filtered based on sequencing quality, mapping quality and mismatch rates. Mutations are removed if their frequencies are lower than a certain threshold (default 0.1). The frequencies of each site of the genome are calculated by an internal function *BSLevel*. The formulae to calculate the frequencies are listed in Table S1. For the remaining candidate SNP set, positions, reference bases, the numbers of supporting reads and average sequencing quality for all four bases in both Watson and Crick strands are recorded (see example below). In order to improve memory and computation efficiency, these data are dynamically allocated and freed for each chromosome. For each position in a chromosome, the data are stored in the form of a vector; thus the data of a chromosome are stored in a matrix. The content and size of the matrix change with the chromosomes. The method is thus called “dynamic matrix algorithm”.

Example of recorded information in this step (Note that frequencies are not recorded):

| Chr | Pos | Ref | Watson (#A,T,C,G) | Crick (#A,T,C,G) | Watson (mean Qual A,T,C,G) | Crick (mean Qual A,T,C,G) |
| --- | --- | --- | --- | --- | --- | --- |
| chr1 | 10583 | G | 70,0,0,24 | 0,2,0,243 | 33,0,0,32 | 0,35,0,33 |

*Step 2. Approximate Bayesian modeling.* In brief, the Bayesian inference of each genotype is based on its posterior distributions, *P*(*G*|*D*), using Bayes’ formula. The posterior distribution is built upon two components: the prior distribution of each genotype *P*(*G*), and the likelihood *P*(*D*|*G*), which is the probability of observing reads *D* given genotype *G*. For the prior *P*(*G*), we referred to the model of SOAPsnp. We observed that, when sequencing depth was higher than 10 (normally available coverage for BS-Seq in sequencing covered regions), the choice of the prior actually had no large effects. The likelihood, which represents the error rates caused by multiple sources, is calculated by the formula *P*(*D*|*g*=*G*)= for multiple independent samples *D_i_*, where *i*=1, 2, …, *n*, and *n* is the number of reads. An internal function *BSStat* performs these calculations. We used average error rate instead of full evaluation of error rates, which greatly increases the modeling speed. The probabilities of all possible ten genotypes (i.e. AA, AT, AC, AG, TT, TC, TG, CC, CG and GG) are calculated. The genotype with largest probability is recognized as the final SNP for the locus.
